# Psychiatric Emergencies in the Community: Characteristics and Outcome in Switzerland

**DOI:** 10.1007/s10488-021-01117-7

**Published:** 2021-02-20

**Authors:** Sonja Moetteli, Raphael Heinrich, Matthias Jaeger, Camillo Amodio, Jan Roehmer, Anke Maatz, Erich Seifritz, Anastasia Theodoridou, Florian Hotzy

**Affiliations:** 1 Department for Psychiatry, Psychotherapy and Psychosomatics, University Hospital of Psychiatry Zurich, Zurich, Switzerland; 2 University of Zurich, Zurich, Switzerland; 3 Psychiatry Baselland, Liestal, Switzerland; 4 SOS-Aerzte, Turicum AG, Zurich, Switzerland

**Keywords:** Psychiatric emergencies, Community services, Crisis intervention, Coercion, Involuntary admission, Hospital admission

## Abstract

Psychiatric emergencies occur frequently in the community setting, e.g. the patient’s home or public places. Little is known about the characteristics and outcome of these situations. This study describes psychiatric emergencies in the canton of Zurich, Switzerland, and examines determinants of their outcome. We retrospectively analyzed 620 medical records of consultations classified as psychiatric emergencies of a 24/7 service of community-based emergency physicians. Information on sociodemographic, clinical and situational factors was extracted. The observation period was 6 months in 2017. Binary logistic regression was used to examine predictors for involuntary admissions. Most emergency consultations (64.5%) took place at the patient’s home, followed by police stations (31.0%), public places (3.2%), and somatic hospitals (1.3%). Patient characteristics and reasons for consultation varied considerably between the locations. The first involved person was commonly a relative. Of all consultations, 38.4% resulted in involuntary admissions, mainly in patients with psychosis, suicidality, aggression, refusal of necessary treatment and previous involuntary admissions. Situation-related factors and the involvement of relatives were no significant predictors of the outcome. Psychiatric emergencies occur in different places and in patients with a variety of psychiatric symptoms. Although half of the emergency situations were resolved in the community, the rate of involuntary admissions was still high. For additional reduction, the further development of quickly available alternatives to psychiatric inpatient treatment is required. These should be specifically geared towards acute situations in patients with the described risk factors. Additionally, the role of relatives during psychiatric emergencies should be further studied.

## Introduction

Psychiatric emergencies (PE), such as acute exacerbations of psychotic or manic illness, suicidal behavior and other acute crises often arise in the community (e.g. at home, in public, at work). These situations require a fast assessment, but a specialized mental healthcare worker (e.g. psychiatrist) is not always available. Therefore, also hospital physicians, general practitioners (GP) or other health professionals, such as paramedics had been described to be responsible for the first assessment and the decision about the next steps (Downey et al. 2012; Fuglseth et al. 2016; Fulbrook & Lawrence 2015; Lally et al. 2015; Rotvold & Wynn 2015). In some PE, a single consultation by a professional or the initiation of psychiatric treatment in the community might be sufficient (Gater et al. 1991). In other situations, more intensive treatment is needed, and patients are immediately referred to inpatient services. At best, this happens on a voluntary basis, but in some cases, if no less intrusive options are available, patients are admitted involuntarily (Marty et al. 2018; Rotvold & Wynn 2015). Involuntary admissions (IA) are exerted when psychiatric treatment is deemed necessary (usually because of harm to self or others due to a psychiatric condition), but is refused by the patient (Lay et al. 2012; Szmukler 2020; Zinkler & Priebe 2002). Due to the restriction of personal freedom, IA are regulated by law (Dressing & Salize 2004; Zhang et al. 2015). Ethical considerations, structural and cultural factors, as well as different legal regulations have been discussed in attempts to understand the variance in IA rates between and within countries (Fiorillo et al. 2011; Lauber & Roessler 2007).

In Switzerland, the exertion of coercion is regulated by the Swiss civil code (Federal Assembly of the Swiss Confederation 2020). The 26 cantons (states) of Switzerland are entitled to adapt parts of the Swiss civil code to their existing structures. In contrast to other cantons with more restrictive regulations, the canton of Zurich (with approximately 1.5 million inhabitants) rules that, besides the Child and Adult Protection Services (Kindes- und Erwachsenenschutzbehörde, KESB), all physicians who are licensed to practice medicine in Switzerland are authorized to order an IA. It has to be made to a “suitable institution”; this is mostly a psychiatric or somatic hospital, but can also be a nursing home.

In modern psychiatry, treatment in the community is usually favored over hospitalization. During PE in the community, the physicians in charge are often alone and might have to find solutions for serious situations in which fast assessment and resolution are needed. In such situations, the physicians might experience internal and external pressures and time constraints. For instance, physicians who referred patients involuntarily often stated to have experienced pressure from third parties such as relatives or the police to initiate the IA (Hotzy et al. 2019b; Rotvold & Wynn 2015). The patient’s environment might favor IA because of desperation, doubts on their ability to care for the patient and associated burdens (Ostman et al. 2000; Weimand et al. 2011). However, whether the involvement of the patient’s relatives in the decision–making process of a PE facilitates IA – or, on the contrary, is a possibility to avoid IA – is still unclear (Roessler 2019).

In general, a history of previous IA and a diagnosis of psychotic disorder (particularly the presence of positive psychotic symptoms), as well as factors such as male gender, unemployment, perceived risk to others, reduced insight into illness and reduced adherence to psychiatric treatment have been identified as main risk factors for IA to a psychiatric hospital (Curley et al. 2016; Hotzy et al. 2019a; Hustoft et al. 2013; Ng & Kelly 2012; Riecher et al. 1991; Silva et al. 2018). However, up to now, situational factors such as police involvement during the decision–making process, whilst being described important, have rarely been systematically studied (Walker et al. 2019). Also, little is known about the characteristics and determinants of the clinical decision–making processes during PE in the community setting (Marty et al. 2018).

### Aims of the Study

Firstly, this study aimed to gather knowledge about the characteristics of PE in the community setting. Secondly, we aimed to examine associations between patient- or situation-related characteristics and outcome of the PE (outpatient and voluntary inpatient treatment versus involuntary inpatient treatment). Based on the results of previous research (Curley et al. 2016; Hotzy et al.2019a; Hustoft et al. 2013; Silva et al. 2018; Van der Post et al. 2009; Walker et al. 2019), we expected IA to be predicted by patient factors such as gender, a history of IA, symptoms of psychiatric disorders related to risk of self-harm or harm to others, and lack of treatment motivation. Regarding situational factors, we explored the involvement of other persons such as the patient’s relatives as well as the date, time and place of the consultation. More knowledge about characteristics of PE and their outcome might help to establish recommendations for the handling of such situations by (non-specialized) professionals and to improve community mental healthcare.

## Methods

### Sample

Consultations of mobile emergency physicians (called SOS-Aerzte AG) who visit patients at various places such as at the patients’ homes or in public were retrospectively analyzed.

**Fig. 1:**
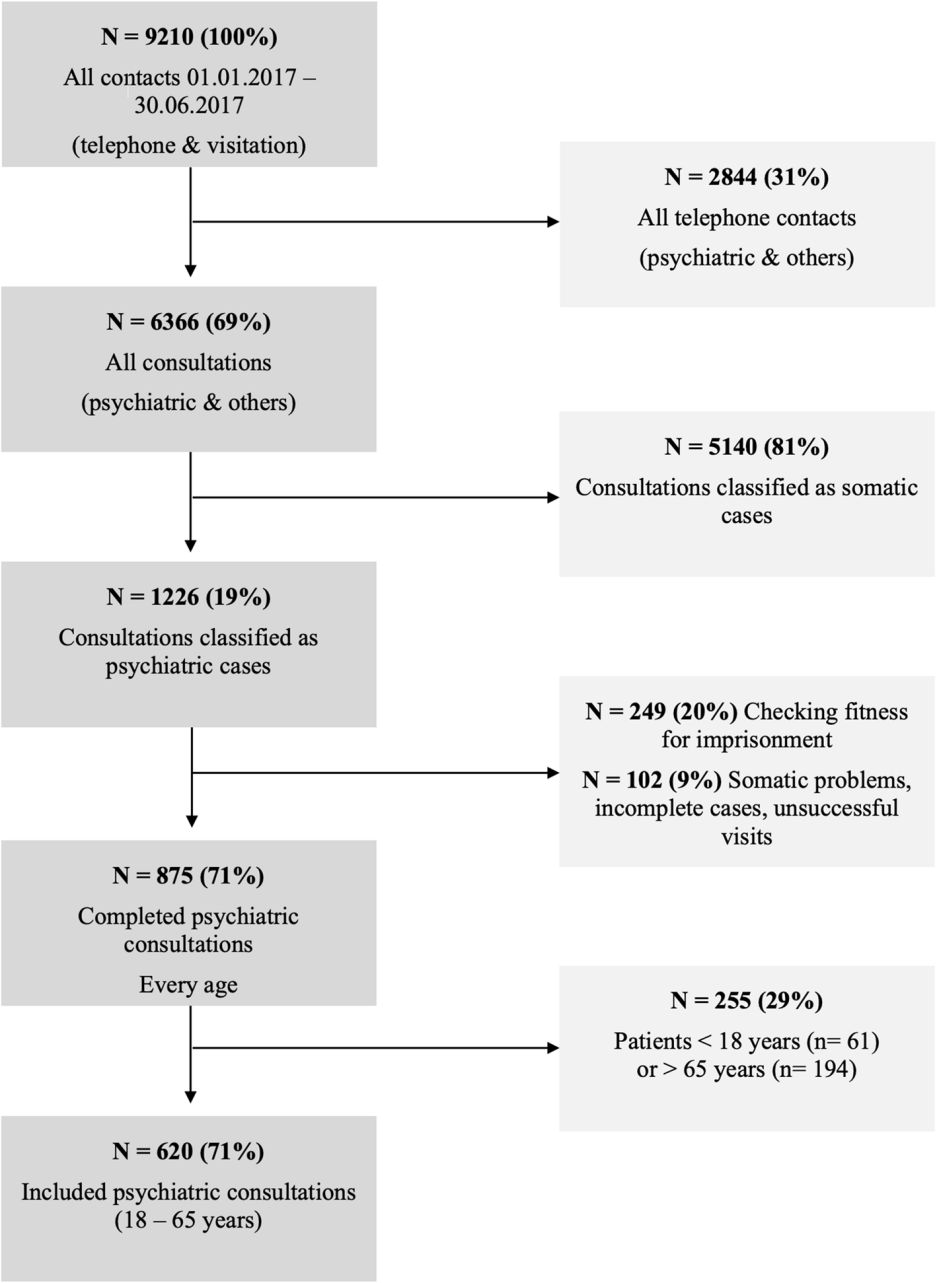
Flow-chart of the selected psychiatric consultations by emergency physicians between January 1st and June 30th 2017

Emergency physicians are responsible for a significant proportion of the outpatient emergency care in the canton of Zurich and previous studies have shown that the quality of their referrals to inpatient care is high (Hotzy et al. 2018; Jaeger et al. 2014; Kieber-Ospelt et al. 2016). Between January 1st 2017 and June 30th 2017, the emergency physicians had *n* = 9210 calls. Approximately one third was dealt with by telephone and *n* = 6366 resulted in face–to–face consultations. We included all consultations for psychiatric problems in persons aged between 18 years and 65 years. We excluded consultations for the assessment of psychological fitness for imprisonment and consultations for somatic problems (for further details see Fig. 1). This resulted in a sample size of *n* = 620.

### Study Design and Procedure

To ensure patients’ anonymity, the emergency physicians developed a standardized data entry form limited to the variables of interest which were retrieved from the patients’ medical records. A psychiatric resident (RH) extracted the patients’ sociodemographic and clinical data as well as situation-related data using a standardized procedure in which the variables of interest and their coding were predefined. Unclear cases were discussed with an emergency physician (CA), a psychiatrist (FH) and a research associate (SM). We conducted a retrospective analysis of the cases identified as psychiatric emergencies (*n* = 620).

### Outcome Parameters

Regarding the patients’ sociodemographic and clinical data, we collected the following variables: gender, age (in years), number of contacts with the emergency physicians in 2017, previous IA, and the presence of critical behavior such as aggression and suicidality. Psychiatric symptoms were grouped into complexes (disorientation/delirium/dementia, intoxication, psychosis/mania, depression, anxiety, personality disorder) adapted from a previous study (Jaeger et al. 2014) and categorized as present versus absent or not applicable.

Regarding situational aspects of the psychiatric emergencies, we collected the following variables: Date of the call (day/month/year), time of the call (between 8:00 am and 6:00 pm = daytime, other = night), place of consultation (1 = the patient’s or a relative’s home, 2 = public place, 3 = police station, 4 = somatic hospital), the person who initiated the consultation (1 = patient, 2 = relative, friend, another third person, 3 = police and security staff, 4 = healthcare professional such as caregiver, nurse, ambulance staff), reason for consultation (1 = evaluation/assessment, 2 = risk of self-harm, 3 = harm to others), number of involved persons, role and order of involved persons (1 = relative, friend, another third person, 2 = police and security staff, 3 = healthcare professional such as caregiver, nurse, ambulance and others, 4 = emergency physician), whether relatives and other persons were involved in the decision–making process (yes versus no), and duration of consultation (minutes).

Regarding the outcome of the consultation, we assessed the following categories: emergency care in the community, referral for voluntary treatment to a psychiatric or somatic hospital, and referral for involuntary treatment to a psychiatric or somatic hospital. In case of referrals to a hospital, we also assessed the type of transport (patient alone or accompanied by healthcare professional, police or relative).

### Statistical Analyses

We descriptively analyzed the characteristics of the psychiatric emergency consultations and compared patient and situational characteristics between the different places of action. Differences in patients’ behavior and symptoms as well as the reasons of consultation were calculated using frequencies and chi-squared tests for multicategorical variables with Bonferroni-adjustment. Differences in the outcome of the PE (IA or voluntary treatment) were calculated using chi-squared tests or *t*-tests for independent samples. Associations were tested using Pearson correlations. To predict the outcome of the PE (IA versus others), we performed a logistic regression with patient characteristics such as gender, age, previous IA, behavior and symptom complex, and situational characteristics such as time of consultation, number of involved persons, time of the emergency physician’s involvement and the relatives’ involvement in the decision–making process. Statistical analyses were performed using IBM SPSS (version 26 for Windows, IBM Corp. 2019). The *p*-value for statistical significance testing was set at alpha ≤ 0.05. All tests were two–sided.

## Results

### Patient Characteristics

Patients’ mean age was 39.9 years (*SD* = 13.1), and *n* = 313 (50.5%) were female. Overall, *n* = 218 patients (35.2%) were already known by the emergency physicians because of previous contacts, and *n* = 318 (51.3%) of all patients had previously experienced IA. Patient characteristics varied between the different places of the emergency consultation (see Table 1).

**Table 1:** Descriptive characteristics of the psychiatric emergency consultations by mobile emergency physicians, differentiated according to the places of action

	All (*n* = 620)	Somatic hospital (*n* = 8)	Patient’s home (*n* = 400)^*^	Public space (*n* = 20)	Police station (*n* = 192)
	% or mean (sd)	% or mean (sd)	% or mean (sd)	% or mean (sd)	% or mean (sd)
Patient characteristics					
Gender, female (vs. male)	50.5	50.0	58.3	55.0	33.9
Age, years	39.9 (13.1)	36.8 (13.7)	40.7 (13.2)	34.8 (13.9)	38.9 (12.7)
Previous IA	51.3	62.5	41.3	35.0	73.4
Call for consultation from					
Patient	16.1	0.0	24.8	0.0	0.5
Relatives and third parties	27.4	0.0	39.5	55.0	0.5
Police & security staff	33.5	0.0	4.8	10.0	97.4
Healthcare professionals & others	22.9	100.0	31.0	35.0	1.6
Reason for consultation					
Evaluation/assessment	50.5	50.0	57.5	65.0	34.4
Risk of self-harm	32.9	37.5	30.5	30.0	38.0
Risk of harm to others	16.6	12.5	12.0	5.0	27.6
Consultation during daytime	58.7	87.5	57.0	85.0	58.3
Duration of consultation, min	54.2 (24.3)	37.5 (13.4)	52.4 (23.5)	57.3 (27.0)	58.3 (25.1)
Persons involved in emergency situation^*^^*^					
Number	2.9 (1.1)	2.6 (0.9)	2.6 (1.1)	2.8 (0.7)	3.5 (0.9)
Emergency physician	100.0	100.0	100.0	100.0	100.0
Relatives and third parties	59.5	37.5	57.3	65.0	64.6
Police & security staff	49.2	12.5	27.3	25.0	100.0
Healthcare professionals & others	62.7	100.0	59.5	70.0	67.2
Relatives involved in decision–making	37.4	25.0	50.2	20.0	13.0
Outcome of situation					
Psychiatric care in the community	42.1	25.0	50.5	65.0	22.9
Voluntary admission to psychiatric hospital	8.2	12.5	8.8	5.0	7.3
Involuntary admission to psychiatric hospital	38.4	62.5	30.0	10.0	57.8
Voluntary admission to somatic hospital	4.5	0.0	5.5	5.0	2.6
Involuntary admission to somatic hospital	6.8	0.0	5.3	15.0	9.4

^*^including relatives’ home, *n* = 30, *IA* involuntary admission(s), ^*^^*^number of involved persons excluding the patient

In nearly a third of all consultations, an adequate conversation was not possible (29.2%). Also, in nearly a third of the consultations, necessary treatment was refused (29.0%). Aggressive behavior was described in *n* = 159 (25.6%) and suicidality in *n* = 112 (18.1%) patients. The most frequently described symptom complex was “psychosis and mania” (34.0%), followed by “intoxication” (28.4%), “depression” (21.8%), “anxiety disorder” (10.8%), and “personality disorder” (10.0%). Patients’ behavior and symptom complexes strongly varied depending on the reason of the consultation (see Table 2).

**Table 2:** Proportion of the patients’ behavior and symptom complexes, differentiated according to the reasons for the psychiatric emergency consultations

	All	Evaluation/assessment	Risk of self-harm	Risk of harm to others		
	% of *n* = 620	*n* (% of *n* = 313)	*n* (% of *n* = 204)	*n* (% of *n* = 103)	Chi-squared value	*p*
Behavior and symptom complexes						
Adequate conversation was not possible	29.2	68 (21.7)a	57 (27.9)a	56 (54.4)b	40.18	< 0.001
Refusal of necessary treatment	29.0	65 (20.8)a	56 (27.5)a	59 (57.3)b	50.52	< 0.001
Aggression	25.6	32 (10.2)c	42 (20.6)a	85 (82.5)b	216.53	< 0.001
Psychosis and mania	34.0	104 (33.2)c	42 (20.6)a	65 (63.1)b	55.30	< 0.001
Intoxication	28.4	65 (20.8)b	89 (43.6)a	22 (21.4)b	34.75	< 0.001
Depression	21.8	51 (16.3)c	79 (38.7)a	5 (4.9)b	57.25	< 0.001
Suicidality	18.1	7 (2.2)b	101 (49.5)a	4 (3.9)b	203.26	< 0.001
Anxiety disorder	10.8	54 (17.3)b	10 (4.9)a	3 (2.9)a	27.53	< 0.001
Personality disorder	10.0	20 (6.4)b	35 (17.2)a	7 (6.8)b	17.32	< 0.001

Identical letters indicate no statistically significant difference by Bonferroni-adjusted chi-squared post-hoc tests; the syndrome complex “desorientation, delirium, dementia” is not listed as total frequency was only 2.1%

### Course of the Consultations

**Fig. 2:**
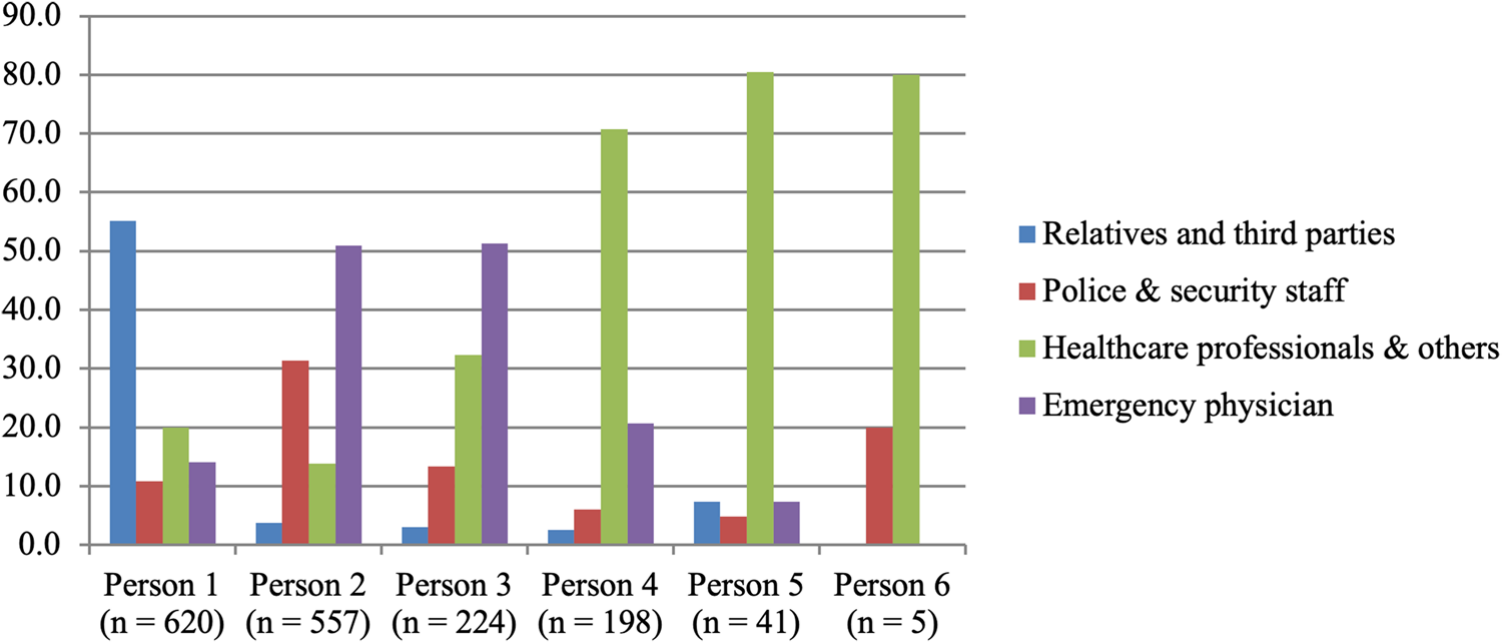
Background of the first to sixth involved person in the psychiatric emergency situation (% of involved groups)

Most consultations (*n* = 400, 64.5%) took place at the patient’s home (including *n* = 30 at their relatives’ home), followed by the police station (*n* = 192, 31.0%), a public place (*n* = 20, 3.2%), and somatic hospitals (*n* = 8, 1.3%). On average, the consultations lasted 54 min and 58.7% took place during daytime. Depending on the place of the consultation, they were initiated either by the patients themselves (16.1%), the patient’s relatives or third parties (27.4), police and security staff (33.5) or healthcare professionals and others (22.9%). Consultations at the patient’s home and in public places were mostly initiated for a clinical evaluation/assessment of the patient. In contrast, at the police station and in somatic hospitals, the reason for consultation was often risk- assessment of self-harm or harm to others. Besides patient and emergency physician, additional persons were involved in most of the consultations. The persons first involved in the PE were most frequently relatives or third parties. The second or third person involved was typically the emergency physician. If police and security staff were involved, they often arrived as second persons. The fourth to sixth persons involved were mostly healthcare professionals such as ambulance staff (see Fig. 2). The results regarding the course of the psychiatric emergency consultations are presented in detail in Table 1.

### Outcome of the Consultations

The two most common outcomes of the emergency consultations were care in the community setting (42.1%) and IA to a psychiatric hospital (38.4%). Only 12.7% of the consultations resulted in a voluntary hospitalization. The proportion of IA was highest if police and security staff initiated the consultation (49.6%). It was comparable for consultations initiated by healthcare professionals (24.3%) and relatives (23.6%), and lowest for consultations initiated by patients themselves (2.5%) (X^2^ = 101.41 (3), *p* < 0.001). Of those who were referred to a hospital, most patients were transported by healthcare professionals/ambulance (54.2%), followed by the police (32.1%) and the patient’s relatives (9.8%). Only 3.4% of the patients went to a hospital by themselves. On average, consultations resulting in an IA took 10 min longer (M_IA_ = 62 min, SD = 24 min, M_Voluntary_ = 47 min, SD = 22 min, t(618) = 8.38, *p* < 0.001). Also, they involved more persons (M_IA_ = 3.6, SD = 0.9, M_Voluntary_ = 2.4, SD = 1.0, t(618) = 16.88, *p* < 0.001). This was true for all groups (relatives, police, and healthcare professionals). The order of the persons involved did not differ between consultations resulting in IA and consultations leading to voluntary treatment. The duration of consultation correlated positively with the number of persons involved (r = 0.31, *p* < 0.001). Consultations with patients who had a history of IA, refused necessary treatment, and with a high risk of self-harm or harm to others (e.g. suicidality, aggression) had a higher likelihood to result in an IA. In contrast, gender was not a significant predictor. Regarding the examined situational factors, only the number of involved persons was a significant predictor of IA while the place (police station versus other places) and the involvement of the patient’s relatives were no significant predictors. Overall, the logistic regression model showed excellent model fit (see Table 3).

**Table 3:** Psychiatric emergency outcomes (IA = 1, other = 0) predicted by patient and situational characteristics, examined by logistic regression (*n* = 620)

	B	sd	OR (95% CI)	*p*
Patient and situational characteristics of emergency situation				
Constant	−9.14	1.43	0.000	< 0.001
Gender (female = 0, male = 1)	−0.73	0.45	0.48 (0.20–1.17)	0.108
Age, years	0.01	0.02	1.01 (0.98–1.05)	0.405
Previous IA (yes = 1, no = 0)	5.30	0.65	199.68 (55.62–716.83)	< 0.001
Adequate conversation was not possible (yes = 1, no = 0)	1.71	0.51	5.50 (2.01–15.04)	0.001
Refusal of necessary treatment (yes = 1, no = 0)	1.40	0.51	4.06 (1.50–10.98)	0.006
Aggression (yes = 1, no = 0)	1.75	0.56	5.73 (1.90–17.24)	0.002
Psychosis and mania (yes = 1, no = 0)	0.93	0.50	2.53 (0.94–6.79)	0.065^*^
Intoxication (yes = 1, no = 0)	−0.18	0.45	0.84 (0.35–2.02)	0.691
Depression (yes = 1, no = 0)	1.21	0.61	3.36 (1.02–11.10)	0.047
Suicidality (yes = 1, no = 0)	2.12	0.59	8.34 (2.63–26.45)	< 0.001
Anxiety disorder (yes = 1, no = 0)	0.50	0.91	1.65 (0.28–9.85)	0.581
Personality disorder (yes = 1, no = 0)	0.36	0.70	1.43 (0.37–5.62)	0.605
Place police station (yes = 1, no = 0)	0.43	0.52	1.53 (0.55–4.26)	0.416
Consultation during daytime (yes = 1, no = 0)	−0.06	0.45	0.94 (0.39–2.25)	0.885
Number of involved persons	0.80	0.32	2.23 (1.18–4.22)	0.013
Timepoint of involvement of emergency physician (1 = earliest to 5 = latest)	0.02	0.40	1.02 (0.47–2.22)	0.962
Relatives involved in decision–making (yes = 1, no = 0)	0.62	0.51	1.87 (0.69–5.07)	0.221
− 2LL	179.78			
Omnibus test	× 2 = 670.72, df = 17, *p* < 0.001			
Nagelkerkes R^2^	0.89			
AUC ROC	0.99			

*IA* involuntary admission(s), *if controlled for self-harm and harm to others, psychosis and mania is also a significant predictor (*p* = 0.037), the significance of the other predictors remained the same

## Discussion

In addition to previous studies on PE which described mostly patients who were referred to a hospital, our study focused on a group of non-psychiatric emergency physicians who provide emergency consultations in the community, such as the patient’s home. Therefore, it provides insights into clinical, but also procedural aspects of these situations which can be resolved in the community in nearly half of the cases. Emergency physicians were shown to be responsible for a relevant number of psychiatric emergency consultations in the canton of Zurich (Kieber-Ospelt et al. 2016) and PE account for about a fifth of their clinical consultations, which is a relevant amount and comparable to other non-psychiatric emergency care settings (Downey et al. 2012; Fulbrook & Lawrence 2015; Lally et al. 2015; Rotvold & Wynn 2015).

In our sample, emergency physicians were called to different places in the community and for different reasons. When the consultation was initiated by institutions such as hospitals or the police, the purpose of the consultation was usually more specific compared to situations in which patients or their relatives were the initiators (more general assessments/clarifications). Whilst most consultations were solved in the community, a substantial part of the consultations resulted in IA. Only a small proportion resulted in a voluntary referral for inpatient treatment. The patient-related risk factors for IA identified in this study are comparable to those described in the previous literature (Curley et al. 2016; Hotzy et al. 2019a; Hustoft et al. 2013; Ng & Kelly 2012; Riecher et al. 1991; Silva et al. 2018): psychosis, suicidality, aggression, refusal of necessary treatment and previous involuntary admissions. Situation-related factors and the presence/involvement of relatives did not significantly influence the outcome of the consultation in this study (i.e. if IA was initiated or not). Previous studies showed that, besides the patients also their relatives feel burdened by the mental illness (Ostman et al. 2000; Weimand et al. 2011) and think that IA should be used for protection more frequently than the patients (Wallsten et al. 2008). Also, physicians who exert IA described pressure of the patients’ relatives to exert IA (Hotzy et al. 2019b; Rotvold & Wynn 2015). Nevertheless, relatives play an important role in the support of patients and are often the first to cope with the patients’ symptoms and express the necessity of IA (Roessler 2019). Further research should focus on the role of relatives as protective but also as driving force regarding IA but also their needs in more detail.

Studies in other non-psychiatric medical settings, such as emergency departments, have shown that mental health problems are relevant in up to 40% of all patients (Downey et al. 2012; Fulbrook & Lawrence 2015; Lally et al. 2015). Due to the high probability to be consulted by patients with (co-morbid) mental health problems in the medical emergency setting, professionals should receive continuous training to deal with the specific challenges of PE: As previous research has demonstrated, professionals typically perceived PE as burdensome, especially when resulting in an IA (Hotzy et al. 2019b; Rotvold & Wynn 2015). To meet the challenges of PE the group of emergency physicians in this study holds regular intervisions (reflective discussion groups with professional peers) and training in the handling of PE.

In this study, more than a third of the PE resulted in an IA (mostly to a psychiatric hospital). The majority of these PE were initiated by the police/at the police station. Nevertheless, the regression analysis showed that the place of the consultation was not a significant predictor for IA. Therefore, the high rate of IA after consultations at police stations appears to be primarily due to the patients’ clinical symptomatology and behavior (e.g. they were significantly more often aggressive). Due to their professional role in our society, police and security staff are often those with early involvement in PE when patients seem threatening to themselves or others in public. Although aggression is not a psychiatric problem per se, it might be a symptom at the advanced stage of a psychiatric crisis. It requires fast and adequate treatment strategies with a focus on calming of the situation, prevention of harm and maintenance of the patients’ dignity.

Future projects should design strategies to engage this specific group of patients at an early stage into voluntary forms of de-escalative outpatient treatment (e.g. home treatment, day- or night clinics, activities organized by mental health services) to establish a good therapeutic relationship and to foster the patient’s integration in the community (Pahwa et al. 2020). One important aspect of such strategies might be a good staff–to–patient ratio which enables intensive care without time-constraints.

The higher number of the involved persons and the longer durations of PE resulting in IA might have several reasons. First, the Swiss civil code requires that physicians, if possible, talk to relatives about their perception of the situation (“*the burden that the patient places on family members and third parties and their protection must be taken into account*” (Federal Assembly of the Swiss Confederation 2020) when deciding about an IA). Second, the clinical assessment might be time consuming due to communication-problems, the patient’s refusal to accept treatment, but also because of anxiety, delusional mistrust or aggressions and the need to assess suicidality carefully. Third, IA is a last resort (ultima ratio). Other options should have been tried and proven to be unsuccessful or unavailable before initiating an IA. In addition, at an organizational level, it can be time-consuming to arrange an admission and the transport (in our study, mostly with an ambulance to a psychiatric hospital) to an adequate institution. However, we found that the duration of the PE was shorter when patients were admitted for inpatient treatment on a voluntary basis compared to those with an IA. This finding supports the first three reasons.

Our study indicates that those physicians who are authorized to initiate IA should have enough time for this process to ensure good clinical practice. This is not always the case and some referring physicians describe time-pressures during initiation of IA (Hotzy et al. 2019a). Financial compensation for physicians in private practice, but also for hospital physicians who might otherwise have to shorten the consultation due to economic pressures, or the installation of specialized services with funding that is independent of health insurances might be approaches to facilitate a profound evaluation and solution of the PE. Having enough time for careful psychiatric assessments might help to avoid unnecessary IA and expensive inpatient treatment episodes mid- and long-term (Stulz et al. 2015). Furthermore, enough time to discuss the decision for an IA with the patients might also be important to reduce the patients’ perceived coercion because physicians might be able to explain the reasons for their decision and thereby make it seem less arbitrary for the patients. In the long term, the reduction of perceived coercion might help to reduce avoidance of psychiatric services in a further crisis (Smith 1995; Swartz et al. 2003).

This study has certain limitations. The study design is retrospective which limits the quality of the data. The emergency physicians did not document their consultations in a completely standardized way. Therefore, clinical information was limited for some cases. The data did not allow to evaluate the physicians’ or patients’ subjective perceptions during and after the consultation. In addition, we only collected data from one group of emergency physicians which limits the generalizability of our findings to other mental healthcare systems with differing cultural, legal and structural backgrounds.

In conclusion, our study shows the great variability of consultations for PE. Previous studies focused on characteristics of patients (Curley et al. 2016; Hotzy et al. 2019a; Hustoft et al. 2013; Ng & Kelly 2012; Riecher et al. 1991; Silva et al. 2018; Walker et al. 2019) and the role of the consulting physicians (Fuglseth et al. 2016; Marty et al. 2018; Rotvold & Wynn 2015) during PE which resulted in (involuntary) hospitalizations. In addition, this study provides information about PE occurring in the community setting (such as the patient’s home), their situational and procedural characteristics and their outcome.

Although the emergency physicians’ provision of on-call visits was helpful to deescalate nearly half of the situations in the community setting, PE still resulted in a high rate of IA. Our results confirmed previous findings of patient-related risk factors for IA and revealed that situation-related factors and the involvement of relatives did not significantly influence the outcome of the consultations. For an additional reduction of IA, the further development of quickly available alternatives to psychiatric inpatient treatment with real time information about treatment capacities and an option to book appointments 24/7 might be helpful. Also, further studies should clarify the role of relatives during PE and in how far their resilience, involvement and relationship with the patient influence the decision for or against IA.
